# Datasets on hub-height wind speed comparisons for wind farms in California

**DOI:** 10.1016/j.dib.2018.05.031

**Published:** 2018-05-18

**Authors:** Meina Wang, Paul Ullrich, Dev Millstein

**Affiliations:** a University of California, Davis, CA, USA; b Lawrence Berkeley National Laboratory, Berkeley, CA, USA

## Abstract

This article includes the description of data information related to the research article entitled “The future of wind energy in California: Future projections with the Variable-Resolution CESM”[1], with reference number RENE_RENE-D-17–03392. Datasets from the Variable-Resolution CESM, Det Norske Veritas Germanischer Lloyd Virtual Met, MERRA-2, CFSR, NARR, ISD surface observations, and upper air sounding observations were used for calculating and comparing hub-height wind speed at multiple major wind farms across California. Information on hub-height wind speed interpolation and power curves at each wind farm sites are also presented. All datasets, except Det Norske Veritas Germanischer Lloyd Virtual Met, are publicly available for future analysis.

**Specifications Table**

**Table:** 

Subject area	*Meteorology*
More specific subject area	*Wind field, wind energy*
Type of data	*Figure, text file*
How data was acquired	*Model simulation, model data post-processing, interpolation*
Data format	*Raw, remapped, analyzed,*
Experimental factors	*Datasets from different sources were analyzed and compared.*
Experimental features	*Hub-height wind speed from different datasets were assessed by comparing to observational data.*
Data source location	*University of California, Davis, Davis, CA, USA*
Data accessibility	*Datasets, except Det Norske Veritas Germanischer Lloyd Virtual Met, are available with this article*
Related research article	*The future of wind energy in California: Future projections with the Variable-Resolution CESM (in press)*

**Value of the data**

• The data descriptions contain the vertical interpolation method to extract wind speed at any specific elevation.
• Data can be used for wind speed assessment and future projections.
• The raw data can be applied to other hub-height wind speed calculation algorithms and procedures for future researches.

## 1 Data

Dataset reported in this article contain hub height wind fields, with special focus on wind farms in California. Two modeling products, three reanalysis dataset, and two observational data are described in the article. The interpolation method for calculating hub-height wind speed is also presented in the article, and can potentially be applied to other studies. Power curves used for calculating wind energy capacity factors at each wind farm location are also provided.

## 2 Experimental design, materials, and methods

### 2.1 VR-CESM (Global climate model product)

Data provided in this article includes two simulations using the Variable-Resolution CESM (VR-CESM) model. CESM version 1.5.5, a fully coupled atmospheric, land, ocean, and sea ice model, was utilized. Both simulations used the F-component set (FAMPIC5), which prescribes sea-surface temperatures and sea ice but dynamically evolves the atmosphere and land surface component models. The atmospheric component model is the Community Atmosphere Model, version 5.3 (CAM5) [2] with the spectral-element (SE) dynamical core [3] in the variable-resolution (VR) configuration. The VR model grid used for this study, depicted in Fig. 2 from the reference article [1], was generated for use in CAM and CLM with the open-source software package SQuadGen [4], [5]. On this grid the finest horizontal resolution is 0.125° (~14 km), with a quasi-uniform 1° mesh over the remainder of the globe. Two simulations were conducted using this grid structure: First, the historical run covers the period from October 1st, 1979 to December 31st, 2000, with first three months discarded as the spin-up period, for a total of 21-years. This historical time period was chosen to provide an adequate sampling of inter-annual variability, to coincide with the time period from the rest of the modeling and reanalysis datasets, and because observed sea surface temperatures (which acted as boundary conditions for the simulation) were only available through 2005. For projecting future wind energy change, our mid-century simulation ran with the “business as usual” Representative Concentration Pathway 8.5 (RCP8.5) [6] from October 1st, 2029 to December 31st, 2050, again discarding the first three months for a total of 21-years. Greenhouse gas (GHG) and aerosol forcing are prescribed based on historical or RCP8.5 concentrations for each simulation. More details on VR-CESM can be found in [7], [8], and the model has been applied to previous studies [9], [10].

### 2.2 DNV GL Virtual Met (Dynamically-downscaled regional model product)

The Det Norske Veritas Germanischer Lloyd (DNV GL) Virtual Met product is derived from a hybrid dynamical-statistical downscaling system based upon the Weather Research and Forecasting (WRF) model and an analog-based ensemble downscaling method. A coarse resolution WRF simulation is run for the entire period to be downscaled, while for only a subset of that period a nested version of the same model is run at high resolution. The period over which the coarse and high-resolution runs overlap is called the training period, while the remaining portion is termed downscaling period. For each time of the latter, the best matching coarse estimates (termed "analogs") over the training period are found. The downscaled solution is then constructed from the set of high-resolution values that correspond to the best matching coarse analogs. This method is based upon Delle Monache et al. [11], [12].

The WRF simulation used telescoping, one-way interacting computational grids. Their respective horizontal grid increments are 20 km and 4 km, with the 4 km grid centered over California. The initial and lateral boundary conditions are specified using MERRA-2. The 20 km grid was run for the entire 01 Jan 1980–31 Dec 2015 period, and generated output every hourly, while the nested 4 km grid was run only during the last year of the full simulation (01 Jan 2015 to 31 Dec 2015). The high resolution downscaled dataset is constructed for the entire 36-year period using the 4 km resolution training data and the 20 km simulation (both from the same WRF model configuration). The result is an hourly time series at each 4 km grid point for January 1st 1980 to December 31st 2015. Wind speed and direction at hub heights, including 50 m, 80 m, 140 m, are output. DNV GL served solely as a data provider, and is not responsible for any results from this data.

### 2.3 MERRA-2 (Reanalysis product)

The Modern-Era Retrospective analysis for Research and Applications, Version 2 (MERRA-2) is a reanalysis product for the satellite era using the Goddard Earth Observing System Data Assimilation System Version 5 (GEOS-50) produced by Global Modeling and Assimilation Office (GMAO) at NASA [13]. MERRA-2 integrates several improvements over the first version MERRA product [14]. For the fields used in this study, the spatial resolution is ~55 km with 3-hourly output frequency from 1980 to present. Vertical interpolation of MERRA-2 data was performed to calculate hub height wind speed. Variables used in vertical interpolation were extracted from two subsets: 3-hourly instantaneous pressure level assimilation [15] and hourly instantaneous single level assimilation [16] (extracted at 3-hourly frequency).

### 2.4 CFSR (Reanalysis product)

The Climate Forecast System Reanalysis (CFSR) from NCEP (National Centers for Environmental Prediction) is a global, coupled reanalysis that spans from 1979 to present, with ~55 km spatial resolution and 6-hourly temporal resolution of relevant wind fields [17]. Notably, this temporal resolution is the lowest out of the five datasets used. The analysis subset was chosen for vertical interpolation at 6-hourly frequency.

### 2.5 NARR (Reanalysis product)

The North American Regional Reanalysis (NARR), another NCEP reanalysis product, features a slightly higher spatial resolution of ~32 km. It is a dynamically-downscaled data product with spatial coverage over North America, with 3-hourly temporal resolution from 1979 through present [18]. Hub height wind speeds from NARR were also calculated at this frequency.

### 2.6 ISD (In-situ observations)

The Integrated Surface Database (ISD) from NOAA's National Centers for Environmental Information (NCEI) were used for assessment of hourly 10 m wind speed from model and reanalysis. The ISD observational stations are distributed globally, with the highest concentration of stations found in North America. Stations across California that provide full year data were selected. As not all stations had continuous temporal coverage between 1980 to 2000, each year was calculated separately so as to maximize the number of available stations. To compare 10 m wind speeds from model and reanalysis datasets to ISD, the nearest grid point values to each of the ISD stations was used. Coastal stations were neglected in the analysis of 10 m winds, due to coastal biases that tend to occur in near-surface coarse-resolution reanalysis. These biases tend to emerge because similarity theory is typically employed to extract 10 m wind speeds, which produces distinctly different results over the ocean and land surface.

### 2.7 Upper air soundings (In-situ observations)

Upper air soundings (vertical wind profiles) from all the available locations across California are incorporated into the comparison (University of Wyoming, Department of Atmospheric Science (http://weather.uwyo.edu/upperair/sounding.html). The three available sounding locations in California are OAK at Oakland airport (station number 72493), VBG at Vandenberg Air Force Base (72393), and NKX at San Diego (72293). The time period from the first two stations spans 1980 to 2000. NKX only has data available starting from September 1989, so only the full years 1990–2000 were assessed. Soundings were collected at 12 hourly intervals at 00Z and 12Z, and logarithmic vertical interpolation was performed to calculate hub-height wind at each sounding location. However, this logarithmic interpolation from sparsely sampled profile data could introduce uncertainties into the calculation.

### 2.8 Wind speed interpolation method

**Fig. 1:**
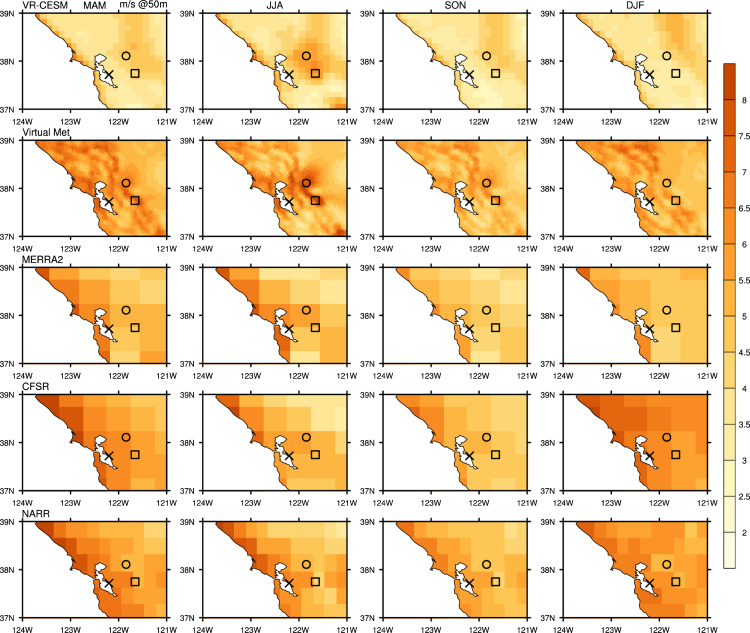
Seasonal average of interpolated 50 m wind speed from each dataset for historical time period 1980–2000 in northern California domain.

**Fig. 2:**
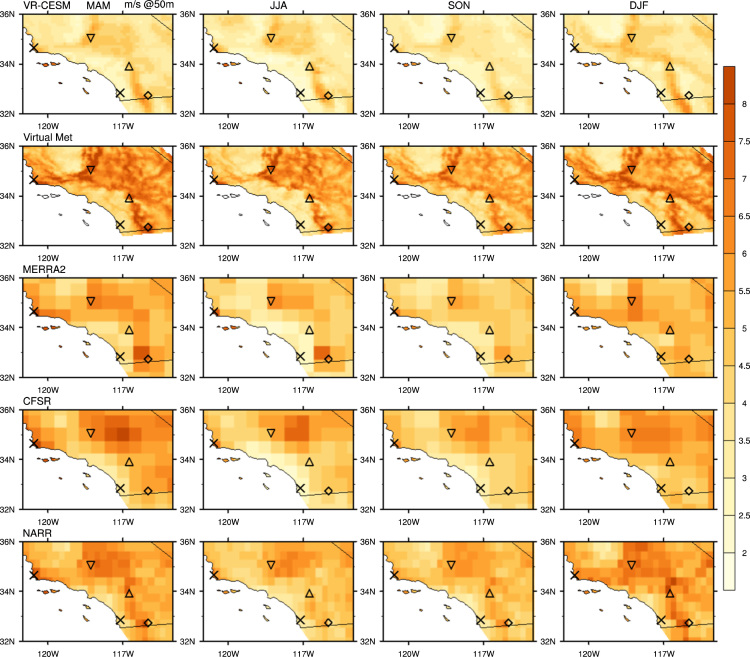
Seasonal average of interpolated 50 m wind speed from each dataset for historical time period 1980–2000 in southern California domain.

**Fig. 3:**
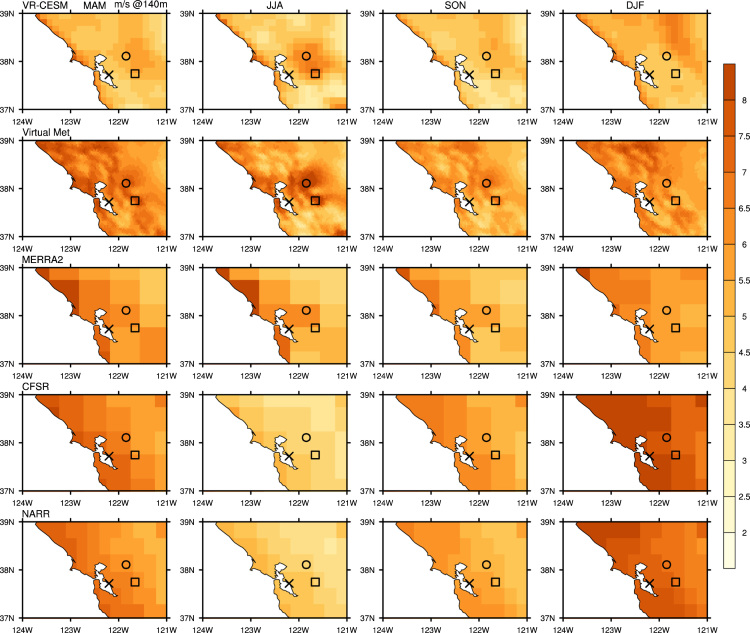
Seasonal average of interpolated 140 m wind speed from each dataset for historical time period 1980–2000 in northern California domain.

**Fig. 4:**
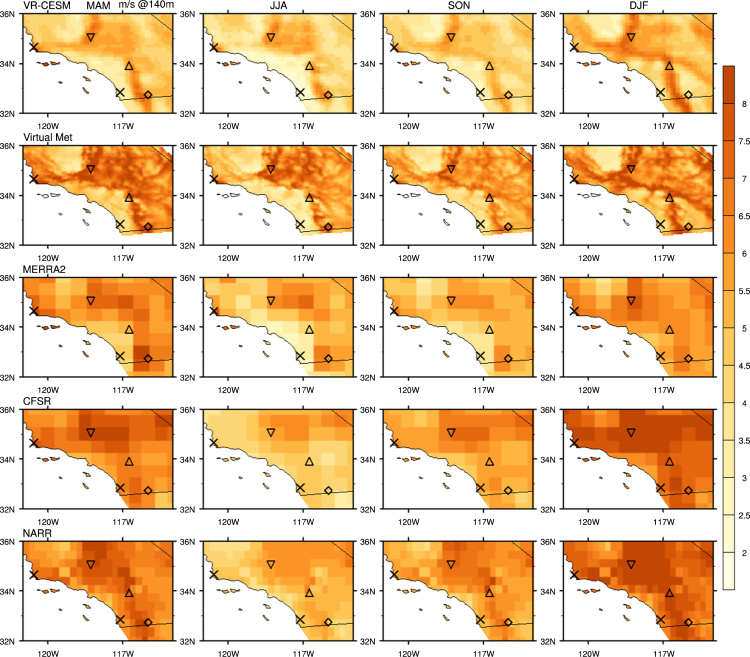
Seasonal average of interpolated 140 m wind speed from each dataset for historical time period 1980–2000 in southern California domain.

The wind speed at each wind farm location (Fig. 1 from the reference article [1]) was determined using nearest grid point values to each wind farm site. To obtain hub-height wind vectors, vertical interpolation was performed on 3-hourly VR-CESM, 3-hourly MERRA-2, 6-hourly CFSR, and 3-hourly NARR products from 1980 to 2000. As mentioned above, hub-height wind output is available directly from the DNV GL Virtual Met data product. Vertical interpolation of VR-CESM data uses the 3D wind field on hybrid surfaces and 10 m altitude wind speed, which is computed from similarity theory. For VR-CESM data, the interpolation procedure is as follows: (1) the CAM5 hybrid coordinates are first converted to pressure coordinates within the column being analyzed, (2) the height of each pressure surface above ground level (AGL) is computed by subtracting the surface geopotential height from the geopotential height of the model level, (3) two model levels that bound the desired interpolation altitude are selected or, if the interpolation altitude is below the lowest model level, the lowest model level and 10 m wind speed field are used, and (4) logarithmic interpolation is applied to obtain the wind speed at the desired interpolation altitude. The interpolation was done by fitting a log equation with the two levels bounding the altitude to be calculated, then with the log profile, interpolating the wind at desired altitude [19]. Vertically interpolated wind speed from MERRA-2, CFSR, NARR, and sounding observations all followed a similar procedure, and were calculated at three hub heights (50 m, 80 m, and 140 m). Fig. 1, Fig. 2, Fig. 3, Fig. 4 show the interpolated hub-height wind speed at 50 m and 140 m, respectively, at northern and southern California. For wind speed at 80 m, and further wind speed analysis, please refer to the cosubmitted research article [1].

Wind turbines can contribute to energy via the electric power system. This contribution is the total amount of usable energy supplied by the turbine per year [20]. The capacity factor (CF) is often defined as actual power output divided by the max amount of wind power that can be generated through the system. This wind speed and CF relationship is not continuous since there is a discontinuous minimum and maximum wind speed required to begin and cease wind power production (the latter to avoid damage to the wind turbine under extreme wind conditions), and this is represented with different power curves associated with each of the wind farm sites. The calculated CF at each wind farm site is based on different characteristic power curves at that site, and do not include electrical losses during the power generation process. The normalized power curves at each wind farm sites, with each value corresponding to a 1 m/s wind speed bin increment starting from 0 m/s, are listed in Table 1. To calculate the CF, wind speed is multiplied with the corresponding power curve value from the corresponding wind speed bin, and then times 100 to convert the percentage values. For further details on the CF analysis, please refer to [17].

**Table 1:** Power curves for wind farms across California. Each value corresponds to a 1 m/s wind speed bin increment starting from 0 m/s.

Wind farm	Power curve
San Gorgonio	IECclass1=(0, 0, 0, 0.0043, 0.0323, 0.0771, 0.1426, 0.2329, 0.3528, 0.5024, 0.6732, 0.8287, 0.9264, 0.9774, 0.9946, 0.999, 0.9999, 1, 1, 1, 1, 1, 1, 1, 1, 1)
Altamont Pass, Ocotillo	IECclass2=(0, 0, 0, 0.0052, 0.0423, 0.1031, 0.1909, 0.3127, 0.4731, 0.6693, 0.8554, 0.9641, 0.9942, 0.9994, 1, 1, 1, 1, 1, 1, 1, 1, 1, 1, 1, 1)
Alta, Shiloh	IECclass3=(0, 0, 0, 0.0054, 0.053, 0.1351, 0.2508, 0.4033, 0.5952, 0.7849, 0.9178, 0.9796, 1, 1, 1, 1, 1, 1, 1, 1, 1, 1, 1)
